# Implementation of the *Activate* injury prevention exercise programme in English schoolboy rugby union

**DOI:** 10.1136/bmjsem-2020-001018

**Published:** 2021-05-04

**Authors:** Craig Barden, Keith A Stokes, Carly D McKay

**Affiliations:** 1 Department for Health, University of Bath, Bath, UK; 2 Rugby Football Union, Twickenham, London, UK; 3 Centre for Motivation and Health Behaviour Change, University of Bath, Bath, UK

**Keywords:** injury, sport, rugby, prevention, adolescent

## Abstract

**Objectives:**

The implementation of the *Activate* injury prevention exercise programme has not been assessed in an applied context. This study aimed to (1) describe the knowledge and perceptions of school rugby coaches and players towards injury risk, prevention and *Activate* and (2) evaluate *Activate* implementation in schoolboy rugby using the reach, effectiveness, adoption, implementation and maintenance framework.

**Methods:**

Bespoke electronic surveys were administered to coaches (including support staff) and players at participating English schools (2018–2020). Most questions and statements were answered using a 7-point Likert scale. At baseline, participants detailed their *Activate* awareness and perceptions of injury risk and prevention in schoolboy rugby. At postseason, participants reported *Activate* use throughout the study and their perceptions towards the programme.

**Results:**

At baseline, significant differences existed between coaches (n=106) and players (n=571) in *Activate* awareness (75% and 13% respectively; χ^2^=173.5, p<0.001). Coaches perceived rugby had a significantly greater injury risk than players, while holding more positive perceptions towards injury prevention. At postseason, coaches reported greater *Activate* adoption compared with players (76% and 18% respectively; χ^2^=41.8, p<0.001); 45% of players were unaware if they used the programme. Median session adherence was twice weekly, with a median duration of 10–15 min. This suggests *Activate* was not implemented as intended, with recommendations of three 20 min sessions per week. Both groups identified common barriers to implementation, such as lack of time and inclusion of a ball.

**Conclusion:**

Coaches are instrumental in the decision to implement *Activate*. Targeting behavioural change in these individuals is likely to have the greatest impact on intervention uptake.

**What is already known**

- *Activate* is efficacious in reducing injury risk in English schoolboy rugby union
- *Activate* implementation has not been evaluated

**What are the new findings**

- Coaches reported significantly greater baseline *Activate* awareness than players (75% and 18%, respectively).
- Coaches had significantly greater *Activate* adoption during the study period (76% and 13%).
- Coaches appear to be critical in the adoption and delivery of *Activate* in a school rugby environment.
- Focus on behavioural change in coaches will likely have the greatest effect of *Activate* implementation. Addressing coach barriers and using behavioural change theories may aid this.

## Introduction

The Rugby Football Union (RFU), England’s rugby union governing body, has been championing the *Activate* injury prevention exercise programme. The 20 min warm-up, designed to be completed prior to training and matches, has shown to be efficacious in reducing youth rugby injury risk.^1^ There are three age group-specific programme available, under-15/16/18, incorporating balance, resistance and plyometric exercises with four progressive phases to be completed throughout the season.^1 2^ In a randomised controlled trial of English schoolboy rugby (under-15 to under-18 years old), a 72% reduction in overall match injuries and a 59% reduction in concussions were reported in teams maintaining full compliance through a season (≥3 times per week). However, only 16% of teams in the intervention arm completed *Activate* as prescribed. If highly resourced schools, supported by a research team, could not maintain compliance over a single season, it raises questions regarding *Activate’*s longer-term effectiveness given the complexity of implementing such interventions in broader sporting contexts.^3 4^

Injury prevention programmes across various sports have been impacted by poor implementation.^5–7^ The *11+* (previously *FIFA 11+*) is perhaps the most widely evaluated programme, with meta-analyses revealing a 20%–70% reduction in injury rates across various settings.^8–10^ However, in 2015, only 10% of national football associations endorsed the programme.^11^ Low end-user awareness and adoption have been reported worldwide,^12–14^ highlighting the difficulty in successfully disseminating and implementing such interventions.^4^ Numerous contextual complexities influence the transfer of findings from research to practice, including individual perceptions, social influences, political pressures and physical demands.^15–17^ Many of these factors are not evaluated in research or addressed in practice, possibly due to the misconception that people will automatically adopt efficacious interventions because injury prevention is of high priority.^18 19^

Evaluating influences on end-user behaviour is a critical step towards successful implementation.^3^ This is particularly important in community-based environments where users may be volunteers, lack adequate training or are constrained by time and resources.^20^ One tool used to evaluate the implementation of public health interventions is the reach, effectiveness, adoption, implementation and maintenance (RE-AIM) framework.^21^ Briefly, the framework assesses an intervention through five dimensions (table 1); reach (R), effectiveness (E), adoption (A), implementation (I) and maintenance (M), with barriers and facilitators occurring at each dimension. Sport-specific modifications have been recommended to the original framework,^22^ including evaluating each dimension at different hierarchical levels (eg, coaches and players) because differences in knowledge, perceptions and contextual factors at different levels can influence intervention implementation. This was highlighted in a population of South African schoolboy rugby coaches and players, where awareness and knowledge of the *BokSmart* injury prevention programme significantly differed between these two groups.^23^ RE-AIM suggests that for interventions to have their desired impact, they need to be well known, adopted and implemented over prolonged periods. This is relevant for sports injury prevention programmes,^1 24^ yet research heavily focuses on effectiveness with little assessment of the remaining dimensions.^25 26^ Only efficacy has been assessed for *Activate* in school rugby.^1^

**Table 1:** RE-AIM dimension definitions

Dimension	RE-AIM definition^21^	Operationalised definition
Reach	Proportion of target population that participated in the intervention	Percentage of coaches and players (end-users) aware of *Activate*
Effectiveness	Success rate if implemented as intended	Perception that *Activate* reduced injury risk among end-users
Adoption	Proportion of settings and practices adopting the intervention	Percentage of coaches self-reporting using *Activate* (adoption and delivery to players) Percentage of players self-reporting using *Activate*
Implementation	Extent to which the intervention is implemented as intended	Percentage of end-users using *Activate* as intended (adherence and fidelity)
Maintenance	Extent to which the programme is maintained over time	Perception that *Activate* could be maintained over multiple seasons Percentage of end-users intending to use *Activate* next season

RE-AIM, reach, effectiveness, adoption, implementation and maintenance.

End-user perceptions influence injury prevention behaviours,^3^ thus evaluating these in school rugby coaches, support staff and players would provide valuable information to aid *Activate* implementation. Therefore, this study’s objectives were to (1) describe and compare baseline knowledge and perceptions of rugby union coaches (including support staff) and players towards injury risk, injury prevention and *Activate* and (2) evaluate *Activate’s* ‘reach’, ‘adoption’, ‘effectiveness’, ‘implementation’ and ‘maintenance’ in English schoolboy rugby.

## Methods

### Prestudy *Activate* implementation

Following publication of an efficacy study in July 2017,^1^ the RFU began disseminating *Activate* through online resources and coach development events, offering free regional training workshops for coaches and support staff registering their interest on the RFU website. In 2018, regional workshops were replaced by a ‘workshop on request’ system and all online resources became openly available and immediately downloadable on the website with no need to register. School coaches were free to take part in these activities, but schools were not specifically targeted through advertising campaigns or workshop deliveries prior to the 2018 season. *Activate* dissemination and implementation was completed by the RFU. No information is available regarding the number of website registrations or workshops run by the RFU external to this study.

### Recruitment

The research team compiled a comprehensive, but not exhaustive, database of English schools (n=289). School names were retrieved from the RFU website for those participating in under-12 to under-19 competitions. Email addresses were obtained for school rugby staff members whom possibly influenced team warm-up procedures (directors/heads of rugby, assistant coaches, medical staff, conditioning staff). Additionally, the RFU publicised the study through coach correspondence and social media to aid recruitment, directing potential participants to contact the research team. School rugby seasons started between July and September and finished between December (generally independent schools) and April (government-funded state schools). Recruitment emails were sent inviting schools to join the project in preseason of two consecutive seasons (July–September 2018 and 2019). If a response to the initial recruitment email was not received, a follow-up email was sent 2 weeks later, after which it was accepted that the school did not wish to participate.

At participating schools, a gatekeeper (primarily the coach) was sent electronic links (https://www.onlinesurveys.ac.uk/) to information sheets and consent forms to forward onto team staff (hereby referred to as coaches), players and their parents/guardians. Patients and public were not involved in the study design.

### Baseline measures

Participants were asked to complete an online baseline survey detailing: (A) demographics, (B) perceptions of injury risk in rugby, (C) perceptions of injury prevention in rugby and (D) *Activate* awareness (online supplemental file 1). The coach survey included 26 questions. A refined player survey (13 questions) was used to maximise response rates, containing questions that were reworded to enable comprehension by the youngest participants (Flesch reading ease score=6.7).

10.1136/bmjsem-2020-001018.supp1 Supplementary data

Questions in sections B, C and D were taken from studies investigating end-user perceptions and intentions towards the *11+*.^12 18 27^ These studies evaluated face and content validity of the survey. Questions were reworded to ask about rugby and *Activate*, rather than soccer and the *11+*. These amendments were face validated by the research team prior to administration. *Activate-*specific questions were aligned with the relevant RE-AIM dimensions, using the operationalised definitions presented in table 1 to facilitate interpretation. The survey consisted of single answer multiple choice questions, multiple answer multiple choice questions and scale/rank questions. Scale/rank questions were answered on a 7-point Likert scale, for example *‘strongly agree’* to *‘strongly disagree’*. To prevent bias towards the left of the scale,^28^ Likert scales were reversed randomly throughout.

*Activate* was not mentioned in recruitment correspondence to prevent bias in the ‘awareness’ questions. Gatekeepers were sent a link to the *Activate* website after completing the baseline survey as a coaching resource, but schools were not instructed to adopt the programme.

### Postseason measures

Postseason surveys were administered electronically to coaches and players who completed the baseline survey (online supplemental file 1). These duplicated the baseline survey but contained an additional section (E) investigating *Activate* use (adoption, implementation and maintenance) and perceptions of effectiveness. Facilitators and barriers were investigated with participants selecting multiple-answer prefilled responses if they agreed with the statement provided. This section used questions from previous studies investigating the *11+* 12 18 27 and an unpublished pilot study of *Activate* implementation in men’s community rugby.^29^

### Analysis

Descriptive statistics were used to summarise continuous (mean, SD) and discrete (percentages (%)) participant demographic data. Ordinal data collected from individual Likert scale responses were presented using medians, IQR, percentages (%) and 95% CI. Only participants who reported using *Activate* were included in the analysis of feedback relating to the programme.

Non-parametric Wilcoxon-Mann-Whitney tests used to assess differences between coach and player Likert scale responses. A 2×2 χ^2^ was used to assess differences between groups for dichotomous responses (yes/no; ‘unsure’ responses were excluded from analysis). Statistical significance was accepted at a Bonferroni adjusted α level p≤0.002 (0.05/22 statistical tests) to reduce the risk of type I error.

### Patient and public involvement

Patients and/or the public were not involved in the design, or conduct, or reporting, or dissemination plans of this research.

## Results

### Demographics

Recruitment emails were sent to 289 schools (148 private, 141 state). At baseline, 106 coaches from 31 schools (11%; 25 private, 6 state) and 571 players from 23 schools (8%; 17 private, 6 state) responded to the survey (table 2).

**Table 2:** Participants’ baseline characteristics

Question/demographic	Response	Coaches n (%)	Players n (%)
**School type**	Private (independent)	87 (82%)	393 (69%)
	State (government funded)	19 (18%)	178 (31%)
**Participant age**	Mean Age	37.4 (±10.5)	15.3 (±2.0)
**What is your role?**	Team staff	106 (100%)	–
	Director of Sport	9 (8%)	-
	Head coach/Director of rugby	41 (39%)	–
	Assistant coach	36 (34%)	–
	Team manager	13 (12%)	–
	Conditioning coach	2 (2%)	–
	Medical practitioner	5 (5%)	–
	Player	–	571 (100%)
**If coaching, how many years coaching experience do you have?**			
	Less than 2 years	13 (13%)	–
	2–3 years	10 (10%)	–
	4–5 years	11 (11%)	–
	6+years	65 (66%)	–
**What is the highest level you have coached?**			
	School/club	54 (55%)	–
	Regional junior academy	14 (14%)	–
	County/constituent body	12 (12%)	–
	Divisional	8 (8%)	–
	Professional	3 (3%)	–
	International	7 (7%)	–
**What is the highest coaching qualification you hold?**			
	RFU level 1	16 (18%)	–
	RFU level 2	38 (42%)	–
	RFU level 3	19 (21%)	–
	RFU level 4	5 (5%)	–
	Other	13 (14%)	–
**When did you obtain this qualification?**			
	Less than 2 years ago	20 (26%)	–
	2–3 years ago	12 (16%)	–
	4–5 years ago	18 (23%)	–
	More than 5 years ago	27 (35%)	–
**What age group do you coach/play in?**			
	Under-12/13	16 (13%)	107 (19%)
	Under-14/15	34 (27%)	167 (29%)
	Under-16	9 (7%)	26 (5%)
	Under-18/19	42 (33%)	271 (47%)
	Multiple age groups	5 (4%)	–
**Have you previously played competitive rugby?**			
	No	9 (8%)	–
	Yes	97 (92%)	–
**If yes, what is the highest level you have played?**			
	School	12 (12%)	–
	Age group community club	4 (4%)	–
	Junior academy Rugby	3 (3%)	–
	University	11 (11%)	–
	Adult community club	47 (48%)	–
	Professional	13 (13%)	–
	International	7 (7%)	–
**Do you have a current medical or first aid qualification?**			
	No	30 (28%)	–
	Yes	76 (72%)	–
**Have you ever used a specific programme to reduce your/players injury risk?**			
	No	65 (61%)	401 (70%)
	Yes	41 (39%)	170 (30%)
**In the past 12 months, have you experienced a rugby injury that caused you to miss a game or training session?**			
	No	–	244 (43%)
	Yes	–	327 (57%)
**If yes, did it cause you to miss school or work for at least 1 day?**			
	No	–	218 (67%)
	Yes	–	109 (33%)

RFU, Rugby Football Union.

### Perceptions

Coaches ‘slightly agree*d’* that rugby players are at high risk of injury, believing injuries have negative effects on team performance and long-term player health (table 3). Coaches (51% ‘agreed*’*, 95% CI 41 to 61) held significantly stronger perceptions than players that rugby injuries could be prevented (45% ‘agreed*’*, 95% CI 41 to 49; z=−3.3, p≤0.001). Most coaches ‘strongly agreed*’* that injury prevention exercises should be performed by rugby players, ‘agreeing*’* that a rugby specific warm-up could reduce injury risk while improving players’ physical characteristics.

**Table 3:** Baseline perceptions of coaches and players towards injury risk and injury prevention, percentage responding per answer (95% CI)

Statement…	Role	n	Median	Strongly agree			Neither		Strongly disagree	
			(IQR)	1	2	3	4	5	6	7
Rugby Injuries can…										
…shorten a player’s career	Coach	106	1	74%	16%	6%	4%	0%	0%	0%
			(1−2)	(66−82)	(9−23)	(1−11)	(0–8)	(−)	(−)	(−)
… cause physical problems later in life	Coach	106	1	61%	28%	10%	1%	0%	0%	0%
			(1−2)	(52−70)	(19−37)	(4−16)	(0–3)	(−)	(−)	(−)
… have a negative impact on team performance	Coach	106	2	21%	37%	14%	12%	5%	7%	6%
			(2−4)	(13−29)	(28−46)	(7−21)	(6−18)	(1−9)	(2−12)	(1−11)
… have a negative impact on a player’s quality of life	Coach	106	2	21%	40%	23%	4%	7%	3%	2%
			(2−3)	(13−29)	(31−49)	(15−31)	(0–8)	(2−12)	(0–6)	(0–5)
Rugby players are at high risk of suffering an injury	Coach	106	3	15%	33%	29%	8%	9%	3%	2%
			(2−3)	(8−22)	(25−43)	(19−37)	(3−13)	(4−14)	(0–6)	(0–5)
	Player	571	3	9%	34%	28%	10%	9%	7%	3%
			(2−4)	(7−11)	(30−38)	(24−32)	(8−12)	(7−11)	(5−9)	(2−4)
I expect/a player I coach to sustain an injury sometime during the next season	Coach	105	3	15%	34%	28%	8%	5%	9%	1%
			(2−3)	(8−22)	(25−43)	(19−37)	(3−13)	(1−9)	(4−14)	(0–3)
	Player*	571	3	5%	17%	31%	18%	9%	15%	5%
			(3−5)	(3−7)	(14−20)	(27−35)	(15−21)	(7−11)	(12−18)	(3−7)
It is possible to prevent some rugby injuries	Coach	105	2	36%	51%	11%	0%	1%	1%	0%
			(1−2)	(27−45)	(41−61)	(5−17)	(−)	(0–3)	(0–3)	(−)
	Player*	571	2	26%	45%	22%	2%	2%	3%	0%
			(1−3)	(22−30)	(41−49)	(19−25)	(1−3)	(1−3)	(2−4)	(−)
Exercises which have been shown to prevent injuries should be…										
…performed by rugby players	Coach	106	1	52%	45%	3%	0%	0%	0%	0%
			(1−2)	(42−62)	(36−54)	(0–6)	(−)	(−)	(−)	(−)
	Player	571	2	50%	42%	5%	2%	1%	0%	0%
			(1−2)	(46−54)	(38−46)	(3−7)	(1−3)	(0–2)	(−)	(−)
…incorporated into schools’ rugby training	Coach	106	2	44%	43%	4%	0%	1%	0%	8%
			(1−2)	(35−53)	(34−52)	(0–8)	(−)	(0–3)	(−)	(3−13)
… varied and progressed over time	Coach	106	2	43%	47%	6%	4%	0%	0%	0%
			(1−2)	(34−52)	(37−57)	(1−11)	(0–8)	(−)	(−)	(−)
Completing a rugby specific warm-up programme prior to every game and training session will…										
…reduce the risk of players sustaining an injury	Coach	106	2	35%	48%	15%	2%	1%	0%	0%
			(1−2)	(26−44)	(38−58)	(8−22)	(0–5)	(0–3)	(−)	(−)
	Player	571	2	44%	42%	10%	2%	2%	0%	0%
			(1−2)	(40−48)	(38−46)	(8−12)	(1−3)	(1−3)	(−)	(−)
… improve physical characteristics such as balance, agility and strength	Coach	106	2	29%	48%	12%	4%	2%	1%	4%
			(1−2)	(20−38)	(38−58)	(6−18)	(0–8)	(0–5)	(0–3)	(0–8)

n=number of respondents per statement.

*P≤0.001 when assessing coach versus player responses.

**Figure 1:**
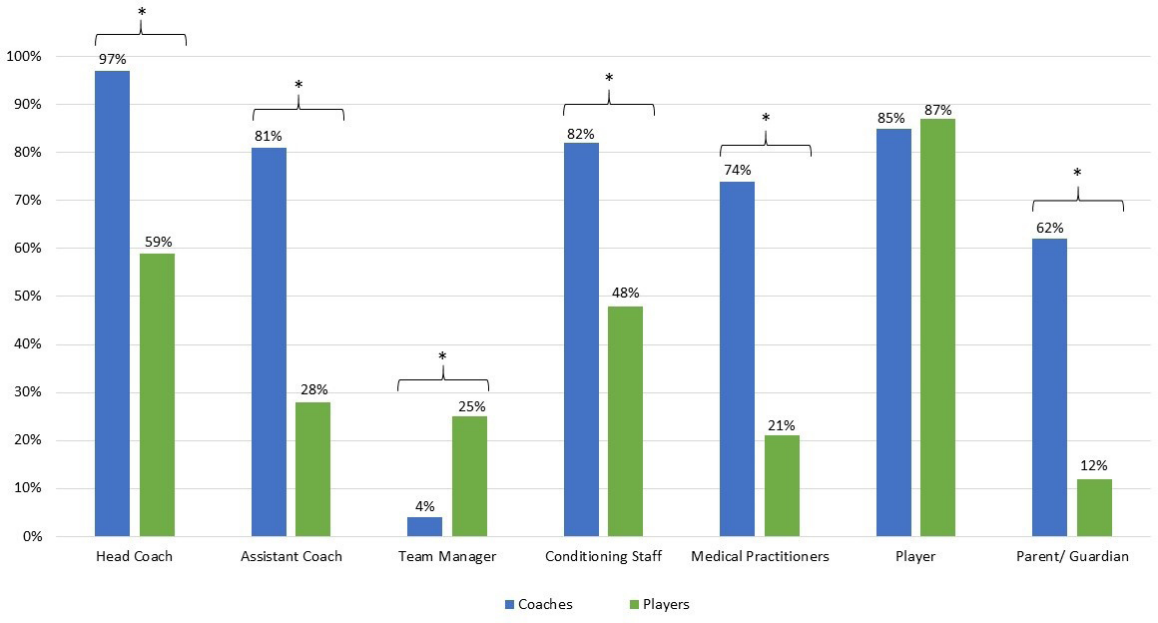
Baseline coach and player response to ‘who is responsible for injury prevention’? *P<0.001 when assessing coach versus player responses.

Significant differences existed between coaches and players when asked ‘who is responsible for injury prevention?’ (figure 1). Both groups rated themselves highest (97%, 95% CI 94% to 100% and 87%, 95% CI 84% to 90%, respectively). Coaches felt injury prevention was a collective responsibility across all roles, except team managers (4%), while players thought responsibility was confined to themselves, head coaches and conditioning staff (all remaining roles <30%).

### Reach and adoption (baseline)

**Figure 2:**
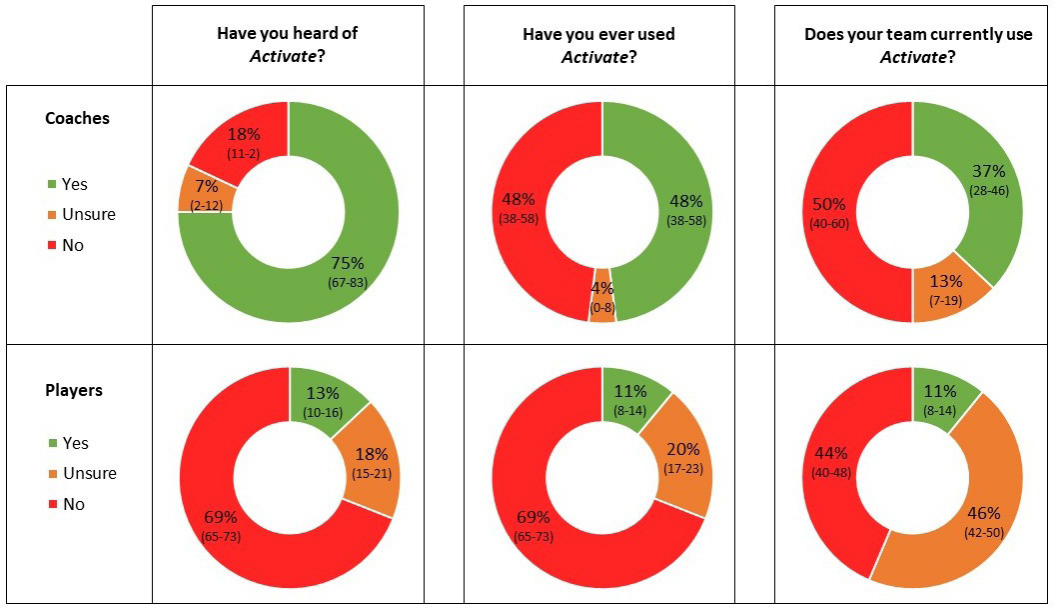
Baseline coach and player responses to *Activate* awareness and adoption. percentage responding per answer (95% CI).

At baseline, most coaches were aware of *Activate* (75%, 95% CI 67% to 83%; figure 2) but fewer than half reported previous (48%, 95% CI 38% to 58%) or current use (37%, 95% CI 28% to 46%). Coach awareness largely came from peers (45%, 95% CI 36% to 54%), the RFU website (43%, 95% CI 33% to 52%) and RFU community rugby coaches (24%, 95% CI 16% to 32%) who were employed by the RFU to support community schools and clubs.

Significantly fewer players were aware of *Activate* at baseline than coaches (13%, 95% CI 10% to 16%; χ^2^=173.5, p<0.001). A small percentage reported previously or currently using *Activate* (both 11%, 95% CI 8% to 14%), with a large proportion unsure if they currently used the programme (46%, 95% CI 42% to 50%). Player awareness mainly came from their coaches (77%, 95% CI 67% to 87%), with all remaining options under 14%.

### Effectiveness (postseason)

Coaches with experience using *Activate* believed it could reduce injury risk (53% ‘agreed*’*, 95% CI 41% to 65%; table 4). Adopting coaches held stronger perceptions it prevented injuries in their team (43% ‘slightly agreed*’*, 95% CI 30% to 56%) than players (41% ‘neutral*’*, 95% CI 28% to 54%; z=−3.3, p<0.001).

**Table 4:** Postseason perceptions from end-users who reported previous *Activate* use. percentage responding per answer (95% CI)

Statement:	RE-AIM	Role	N	Median	Strongly agree		Neither			Strongly disagree	
				(IQR)	1	2	3	4	5	6	7
*Activate* can prevent rugby injuries in your team	E, A	Coach	62	2	26%	53%	15%	3%	0%	0%	3%
				(1−2)	(15−37)	(41−65)	(6−24)	(0–7)	(−)	(−)	(0–7)
*Activate* is rugby specific	A, I, M	Coach	62	3	15%	16%	24%	18%	13%	13%	2%
				(2−5)	(6−24)	(7−25)	(13−35)	(8−28)	(5−21)	(5−21)	(0–5)
		Player	57	4	11%	23%	16%	28%	5%	12%	5%
				(2−4)	(3−19)	(12−34)	(6−26)	(16−40)	(0–11)	(4−20)	(0–11)
*Activate* is too long	A, I, M	Coach	62	4	2%	16%	21%	22%	11%	23%	5%
				(3−6)	(0–5)	(7−25)	(11−31)	(12−32)	(3−19)	(13−33)	(0–10)
		Player	57	4	2%	16%	12%	42%	12%	11%	5%
				(3−5)	(0–6)	(6−26)	(4−20)	(29−55)	(4−20)	(3−19)	(0–11)
*Activate* was fun to do	A, I, M	Player	57	4	5%	11%	21%	42%	11%	3%	7%
				(3−4)	(0–11)	(3−19)	(10−32)	(29−55)	(3−19)	(0–7)	(0–14)
*Activate* contains adequate variation and progression for our team	A, I, M	Coach	62	2	3%	55%	27%	7%	8%	0%	0%
				(2−3)	(0–7)	(43−67)	(16−38)	(1−13)	(1−15)	(−)	(−)
*Activate* could be maintained over multiple seasons by our team	A, I, M	Coach	62	2	16%	58%	23%	3%	0%	0%	0%
				(2−3)	(7−25)	(46−70)	(13−33)	(0–7)	(−)	(−)	(−)
*Activate* reduced my/players injury risk this season	E, A, I, M	Coach	58	3	0%	26%	43%	9%	14%	7%	2%
				(2−4)	(−)	(15−37)	(30−56)	(2−16)	(5−23)	(0–14)	(0–6)
		Player*	54	4	6%	4%	17%	41%	22%	7%	4%
				(3−5)	(0–12)	(0–9)	(7−27)	(28−54)	(11−33)	(0–14)	(0–9)

Percentage responding per answer (95% C).

*P<0.001 when assessing coach versus player responses.

RE-AIM, reach, effectiveness, adoption, implementation and maintenance.

### Adoption

Coaches reported significantly greater adoption rates than players during the study period (76%, 95% CI 66% to 86%; and 18%, 95% CI 14% to 22%, respectively; χ^2^=41.8, p<0.001). Players were largely unaware whether they used *Activate* during the season (45%, 95% CI 39 to 50). All adopting coaches reported using the programme prior to training, though 16% (95% CI 6% to 25%) did not use it prior to matches.

### Implementation

Adopting coaches had a median adherence of two sessions per week (45%, 95% CI 32% to 58%), with 33% (95% CI 21% to 45%) using *Activate* thrice weekly as recommended. Median duration prior to training was 10–15 min (50%, 95% CI 37% to 63%), with 28% of coaches taking 15–20 min to complete *Activate* (95% CI 16% to 40%). Adopting coaches reported median duration prior to matches was 10–15 min (31%, 95% CI 19% to 43%), with a third spending 5–10 min (33%, 95% CI 21% to 45%). Of adopting players, 41% (95% CI 28% to 54%) reported completing two sessions per week (41%, 95% CI 28% to 54%), with 33% (95% CI 21% to 46%) using *Activate* three times per week. There was no difference between coach and player adherence (χ^2^=−0.1, p=0.9).

### Maintenance

Most coaches ‘agreed*’ Activate* contained adequate variations/progressions (55%, 95% CI 43% to 67%) and could be maintained over multiple seasons (58%, 95% CI 46% to 70%); however, 44% (95% CI 46% to 70%) felt it needed to be improved and 47% (95% CI 35% to 59%) suggested their school develop their own version. Coaches had significantly greater intention (43% ‘strongly agreed’, 43%, 95% CI 32% to 55%) to use *Activate* next season than players (54% ‘neutral*’*, 95% CI 48% to 60%; χ^2^=−5.5, p<0.001).

### Facilitators and barriers

Coaches with experience using *Activate* (in this study or previously) perceived its positives to be ‘learning exercises to reduce my players’ injury risk’ (73%, 95% CI 62% to 84%), followed by ‘completing exercises different to usual rugby training’ (65%, 95% CI 53% to 77%). The most commonly reported barrier from coaches was the lack of ball work within the programme (45%, 95% CI 33% to 57%). Nearly a third of coaches (31%, 95% CI 19% to 43%) reported that players disliking *Activate* was a barrier. Some coaches felt *Activate* limited their time to train (29%, 95% CI 18% to 40%), 32% recommending reducing the programmes duration (95% CI 20% to 44%).

There was no consensus from players regarding facilitators to using *Activate*. Commonly reported player barriers were the lack of ball work (37%, 95% CI 24% to 50%) and the resulting lack of time to train (28%, 95% CI 16% to 40%). Only 6% (95% CI 0% to 12%) of players with *Activate* experience said they did not like completing the programme, although 22% (95% CI 11% to 33%) of players reported the exercises were boring.

## Discussion

This study sought to describe the knowledge and perceptions of schoolboy rugby coaches and players towards injury risk, prevention and the *Activate* programme. Coaches had significantly greater perceptions of rugby injury risk and more positive perceptions towards prevention than players. Coaches had high rates of *Activate* awareness and adoption. Only a small percentage of players were aware of the programme, with their awareness largely coming from their coaches. Coaches are critical stakeholders in the decision to adopt and deliver *Activate* in a school context, suggesting implementation strategies should focus on these individuals.

Coaches perceived rugby players were at high risk of injury, agreeing with evidence that injuries can have detrimental effects on team performance,^30^ an athlete’s career^31^ and their quality of life.^31 32^ Coaches and players felt it was possible to prevent rugby injuries, identifying the positive effects rugby specific warm-ups can have on injury risk.^1 33 34^ These findings are encouraging as end-user knowledge and perceptions influence outcome behaviour.^3 35^ However, influences on behaviour are multifactorial^18 19 36^ and the notion that high levels of perceived risk or effectiveness will lead to coaches’ adoption^6^ or adherence^19^ is too simplistic. Altering these perceptions should not be the primary strategy for maximising implementation. Using behavioural change theories may provide success in influencing coach behaviour to maximise outcomes for the latter dimensions of RE-AIM.^18 37^

Using the RE-AIM framework, there was good programme reach among coaches. This is especially positive as this study was conducted within 2 years of *Activate’s* launch and more established programmes have reported poorer coach awareness.^13 14 27 38^ Players had poor programme awareness, likely not affecting their exposure in a school environment but hindering autonomous adoption and long-term maintenance. Coaches reported significantly greater adoption rates than players, many of whom were unaware they were completing *Activate*. This supports the notion that coaches have primary decision-making responsibility and control of injury prevention in youth sport^39^ and directing effort towards behavioural change in these individuals should be a priority. This approach is further advocated given coaches impart their awareness of injury prevention programmes onto their players,^23^ while positively influencing players’ injury prevention behaviours.^40^

Hislop *et al* 1 found greatest efficacy when completing *Activate* three times per week.^1^ Coaches in this present study reported a median adherence of twice weekly. Similar programmes have found significant benefits when used two times per week^41^ so this level of adherence may be sufficient to provide a preventative effect. However, *Activate’s* dose–response relationship needs investigation in future pragmatic trials. Coaches reported a median duration of 10–15 min to complete *Activate*, suggesting the programme was not implemented as intended. Low exercise fidelity in youth athletes, with players not completing all preventative exercises^38 42^ or performing them incorrectly,^42–44^ has been reported in the literature. It is unclear whether the shorter duration noted in this study is related to issues regarding exercise fidelity, but further evaluation is warranted given the potential negative impact on effectiveness.

Prevention programme maintenance is scarcely investigated,^25 26 45^ leaving long-term effectiveness unexplored. Coaches agreed that *Activate* contains adequate variations and progressions to facilitate maintenance, contrasting findings from the *11+* where less than 50% of coaches and players felt the programme could be maintained for multiple seasons.^38^ Uniquely, *Activate* can be progressed over weeks, months and seasons, with each age-specific programme containing four phases. This possibly influenced coaches’ positive perceptions and this approach should be considered when developing future injury prevention programmes.

Reduced training time as a result of completing *Activate* was a reported barrier from coaches and players. Similar barriers restricted *11+* adoption in community football.^18 46^ A recent study found completing *11+* strengthening exercises (part 2) postsession increased adherence without negatively influencing effectiveness.^47^ Before this approach can be advocated for *Activate*, research needs to explore the mechanistic effect of the programme. Certain exercises were included to reduce specific injuries (eg, isometric neck strengthening for concussion). If these exercises induce chronic long-term effects, they could be omitted from the warm-up and completed at a more suitable time. Conversely, if they induce acute physiological effects, they likely need to be completed immediately prior to exposure. Until this is established it would not be appropriate to recommend completing specific parts, or exercises, postsession as a preventative measure.

### Limitations

To mitigate selection bias, the recruitment database was expanded to include 252 additional schools who did not participate in the efficacy study.^1^ In total, 30% of participating schools in this study were involved in the efficacy study. It is unknown if coaches themselves participated in the previous study. At the time, the programme was not called *Activate* and it is unclear if previous participation would have influenced coaches’ awareness or perceptions towards the programme. A large proportion of respondents were from independent schools despite targeting an equal number of state schools in the recruitment process. Beyond school type, no further demographic information is available for non-respondents, reducing the generalisability of the results and increasing the risk of selection bias.

Surveys administered were an amalgamation of those previously used in football^12 18 19 27^ and rugby.^1 29^ They have not been psychometrically evaluated beyond face and content validity. Postseason surveys were completed within 6 months of the end of the season to reduce recall bias.^48^ A 7-point Likert scale was used to minimise the effect of any central tendency bias.^49^ Surveys provided no option for free-text answers. Using qualitative methods may provide greater insight into end-user perceptions and contextual issues.

## Conclusions

This study provides novel findings regarding the implementation of the *Activate* injury prevention exercise programme in English schoolboy rugby. Coaches had significantly greater awareness and adoption of *Activate*, with players largely unaware of the programme and if they used it. Coaches appear key stakeholders in the decision to implement *Activate* in a school rugby environment. Focus on behavioural change in coaches should be a priority to maximise *Activate* uptake.

## Data Availability

All data relevant to the study are included in the article or uploaded as online supplemental information.
