# Supplementary material for: Implementation of the Activate injury prevention exercise programme in English schoolboy rugby union
Source: BMJ Open Sport Exerc Med. 2021 May 4;7(2):e001018. doi: 10.1136/bmjsem-2020-001018 (PMC8098930; doi:10.1136/bmjsem-2020-001018)
Supplement: Supplementary data [file bmjsem-2020-001018supp001.pdf]

Supplementary File 1. Survey questions and responses completed by study participants

Question/ Statement	RE-AIM Dimension	Responses	Coaches		Players	
			Baseline	Post-season	Baseline	Post-season
What is your role?	-	**Select One:**<br>Director of Sport<br>Head coach<br>Assistant coach<br>Team manager<br>Conditioning Coach<br>Medical practitioner<br>Other	A			
If coaching, how many years coaching experience do you have?	-	**Select One:**<br>Never<br>Less than 2 years<br>2-3 years<br>4-5 years<br>6 years+	A			
- What is the highest level you have coached?	-	**Select One:**<br>School/club<br>Regional academy<br>County/constituent body<br>Divisional<br>Professional<br>International	A			
- What is the highest coaching qualification you hold?	-	**Select One:**<br>RFU Level 1<br>RFU Level 2<br>RFU Level 3<br>RFU Level 4<br>Other	A			
- When was this coaching qualification attained (please tick 1 answer)?	-	**Select One:**<br>Less than 2 years<br>2-3 years<br>4-5 years<br>More than 5 years ago	A			
- What age group do you coach/play in?	-	**Select One:**<br>Under-12<br>-<br>Under-19<br>Various	A		A	
When was the last time you completed an official RFU course?	-	**Select One:**<br>Less than 2 years ago<br>2-3 years<br>4-5 years<br>6+ years ago	A			
Have you previously played competitive rugby?	-	**Select One:**<br>No<br>Yes	A			
- If yes, what is the highest level you have played?	-	**Select One:**<br>School<br>Age group community<br>Junior academy<br>University<br>Adult community<br>Professional<br>International	A			
Do you have a current medical or first aid qualification?	-	**Select One:**<br>No<br>Yes	A			
Have you ever used a specific programme to reduce your/players injury risk?	-		A		A	
In the past 12 months, have you experienced a rugby injury that caused you to miss a game or training session?	-				A	
- If yes, did it cause you to miss school or work for at least 1 day?	-				A	
In your opinion, ***how serious*** are the following types of Rugby injuries?<br>- Ankle sprain<br>- Knee ligament sprain<br>- Muscle strain<br>- Concussion<br>- Bone/fracture<br>- Bruise/contusion<br>- Shoulder injury	-	**7-point Likert Scale:**<br>1. Not serious<br>–<br>7. Very Serious				
Rugby injuries can ***shorten a player's career***	-	**7-point Likert Scale:**<br>1. Strongly Agree<br>–<br>7. Strongly Disagree	B			
Rugby injuries can cause ***physical problems later in life***	-		B			
Rugby injuries have a ***negative impact on team performance***	-		B			
Rugby injuries have a ***negative impact on a player's quality of life***	-		B			
Rugby players are at ***high risk of suffering an injury***	-		B		B	
I expect a player I coach to ***sustain a Rugby injury*** sometime during the next	-		B		B	

season						
It is ***possible to prevent*** some Rugby injuries	-		C		C	
Exercises which have been ***shown to prevent*** injuries should be performed by Rugby players	A, M		C		C	
Exercises to prevent injuries should be ***varied and progressed*** over time	A, I, M		C			
Exercises which have been ***shown to prevent*** injuries should be incorporated into Schools Rugby training	M		C			
Completing a Rugby specific warm-up programme prior to every game and training session will ***reduce the risk*** of players sustaining an injury	E, A, I		C		C	
Completing a Rugby specific warm-up programme prior to every game and training session ***will improve*** physical characteristics such as balance, agility and strength	E, A, I		C			
Balance exercises can prevent Rugby injuries	E, A, I					
Controlled jumping/landing exercises can prevent Rugby injuries	E, A, I					
A ***warm-up jog/run*** can prevent Rugby injuries	E, A, I					
Cutting exercises can prevent Rugby injuries	E, A, I					
Cool-down jog/run can prevent Rugby injuries	E, A, I					
Resisted shoulder exercises can prevent ***shoulder injuries*** in Rugby	E, A, I					
Neck muscle strengthening can prevent ***concussion*** in Rugby	E, A, I					
I would like my team to complete a Rugby specific warm-up programme prior to every game and training session next season	A, I, M					
Who is responsible for Rugby injury prevention?<br>- Head coach<br>- Assistant coach<br>- Team manager<br>- Strength and conditioning/ fitness coach<br>- School medical practitioner<br>- Player<br>- Parent/guardian	A, I, M	**Tick all that apply**	C		C	
Had you ***heard of 'Activate'*** before taking part in this survey?	R	**Select One:**<br>Yes<br>No<br>Unsure	D		D	
- If yes, where did you heard about it?<br>- RFU Website<br>- Community Rugby Coaches<br>- Peers<br>- Social media	R	**Tick all that apply**	D		D	
Have you ***ever used*** 'Activate'?	R, A, M	**Select One:**<br>Yes<br>No<br>Unsure	D		D	
Does your team ***currently use*** 'Activate'?	R, A		D	E	D	E
Does 'Activate' need to be ***improved for use*** in your team?	A, I, M			E		
Should your school ***develop its own version*** of 'Activate'?	A, I, M			E		
'Activate' can ***prevent*** Rugby injuries in your team	E, A	**7-point Likert Scale:**<br>1. Strongly Agree<br>–<br>7. Strongly Disagree		E		
'Activate' is ***Rugby specific***	A, I, M			E		E
'Activate' is ***too long***	A, I, M			E		E
'Activate' contains adequate ***variation and progression*** for our team	A, I, M			E		
'Activate' could be ***maintained*** over multiple seasons by our team	A, I, M			E		
Activate' was ***fun to do***	A, I, M					E
In your opinion, what happened to your players' ***overall risk of injury*** as they completed 'Activate' in training or prior to match play playing this season?	E, A, I, M	**7-point Likert Scale:**<br>1. Extremely Increase<br>-<br>7. Extremely Decreased		E		E
How confident are you that ***you understand 'Activate'*** well enough to use it with the School team(s) you are coaching for the upcoming season?	-	**7-point Likert Scale:**<br>1. Extremely Confident<br>-<br>7. Extremely Unconfident				
How confident are you that ***you have the ability to use 'Activate'*** with the School team(s) you are coaching for the upcoming season?	-					
What ***resources were available*** to help you complete 'Activate' with your team at every game and training session during the upcoming season?	-	Free Text Box				
What ***barriers prevented or impeded you*** from the completing 'Activate' with your team at every game and training session during the upcoming season?	-	Free Text Box				
If there was ***limited space*** at your practice or game venue, how confident were you that your team could complete 'Activate'?	-	**7-point Likert Scale:**<br>1. Extremely Confident<br>-<br>7. Extremely Unconfident				
If the players on your team ***did not enjoy*** performing 'Activate', how confident were you that you could still have them complete it at every game and training session?	-					
If you were to start the season by completing 'Activate' with your team at every game and training session, how confident are you that you could continue to do	-					

so for the ***entire season***?						
If you ***missed completing the programme*** at a game or training, how confident were you that you could start completing it again the next session?	-					
On average, how ***many times a week*** did your team complete 'Activate' ***during this season***?	A, I	**Select One:**<br>Never<br><1 x per week<br>1 x per week<br>2 x per week<br>3 x per week		E		E
On average, ***how long did it take*** your team to complete 'Activate' ***during training***?	A, I	**Select One:**<br>We didn't<br>5-10 minutes<br>10-15 minutes<br>15-20 minutes<br>>20 minutes		E		
On average, ***how long did it take*** your team to complete 'Activate' ***before matches***?	A, I			E		
What are some of the things you ***did like*** about doing 'Activate' with your team before training or matches?	A, I, M	**Tick all that apply**:<br>Various prefilled responses		E		E
What were some of the things you ***did not*** like about doing 'Activate' with your team before training or matches (*tick as many as appropriate*).	A, I, M			E		E
What ***changes would you recommend*** to 'Activate' in terms of content or delivery structure	A, I, M			E		
Do you intend to make ***injury prevention a priority*** for your team in the upcoming season?	-	**7-point Likert Scale:**<br>1. Extremely Likely<br>-<br>7. Extremely Unlikely				
Do you ***intend to complete*** 'Activate' with your team at every game and training session in the upcoming season?	A, I, M			E		E
Next season, do you have concrete plans on how to ***teach*** 'Activate' to the players on your School team?	-	**7-point Likert Scale:**<br>1. Strongly Agree<br>–<br>7. Strongly Disagree				
Next season, do you have concrete plans on ***when*** to have your team complete 'Activate'?	-					
Next season, do you have concrete plans on how to ***deal with challenges*** in order to stick to your intentions of completing 'Activate'?	-					
Next season, do you have concrete plans on how to ***encourage your team*** to perform 'Activate' to the best of their ability?	-					

Abbreviation Key:

R = Reach
E = Effectiveness
A = Adoption
I = Implementation
M = Maintenance

Colour Key:

- Green signifies questions asked for this present study, labelled by section…
  - A = demographic
  - B = perceptions towards injury risk in rugby
  - C = perceptions towards injury prevention in rugby
  - D = baseline awareness and use of Activate
  - E = post-season use and perceptions of Activate
- Amber signifies questions asked for a sub-study, which were not included or analysed as part of this study.
